# Estimating the costs and perceived benefits of oral pre-exposure prophylaxis (PrEP) delivery in ten counties of Kenya: a costing and a contingent valuation study

**DOI:** 10.3389/frph.2024.1278764

**Published:** 2024-02-23

**Authors:** Steven Forsythe, Urbanus Kioko, Guy Mahiane, Robert Glaubius, Abednego Musau, Anthony Gichangi, Jason Reed, Daniel Were

**Affiliations:** ^1^ Center for Economics and Costing, Avenir Health, Glastonbury, CT, United States; ^2^ Department of Economics, University of Nairobi, Nairobi, Kenya; ^3^ Center for Modeling, Planning and Policy Analysis, Avenir Health, Glastonbury, CT, United States; ^4^ Jhpiego, Nairobi, Kenya; ^5^ HIV and Infectious Disease Unit, Jhpiego, Baltimore, MD, United States

**Keywords:** HIV, contingent valuation (CV), pre-exposure prophylaxis (or PrEP), Kenya, costing

## Abstract

**Background:**

Kenya included oral PrEP in the national guidelines as part of combination HIV prevention, and subsequently began providing PrEP to individuals who are at elevated risk of HIV infection in 2017. However, as scale-up continued, there was a recognized gap in knowledge on the cost of delivering oral PrEP. This gap limited the ability of the Government of Kenya to budget for its PrEP scale-up and to evaluate PrEP relative to other HIV prevention strategies. The following study calculated the actual costs of oral PrEP scale-up as it was being delivered in ten counties in Kenya. This costing also allowed for a comparison of various models of service delivery in different geographic regions from the perspective of service providers in Kenya. In addition, the analysis was also conducted to understand factors that indicate why some individuals place a greater value on PrEP than others, using a contingent valuation technique.

**Methods:**

Data collection was completed between November 2017 and September 2018. Costing data was collected from 44 Kenyan health facilities, consisting of 23 public facilities, 5 private facilities and 16 drop-in centers (DICEs) through a cross-sectional survey in ten counties. Financial and programmatic data were collected from financial and asset records and through interviewer administered questionnaires. The costs associated with PrEP provision were calculated using an ingredients-based costing approach which involved identification and costing of all the economic inputs (both direct and indirect) used in PrEP service delivery. In addition, a contingent valuation study was conducted at the same 44 facilities to understand factors that reveal why some individuals place a greater value on PrEP than others. Interviews were conducted with 2,258 individuals (1,940 current PrEP clients and 318 non-PrEP clients). A contingent valuation method using a “payment card approach” was used to determine the maximum willingness to pay (WTP) of respondents regarding obtaining access to oral PrEP services.

**Results:**

The weighted cost of providing PrEP was $253 per person year, ranging from $217 at health centers to $283 at dispensaries. Drop-in centers (DICEs), which served about two-thirds of the client volume at surveyed facilities, had a unit cost of $276. The unit cost was highest for facilities targeting MSM ($355), while it was lowest for those targeting FSW ($248). The unit cost for facilities targeting AGYW was $323 per person year. The largest percentage of costs were attributable to personnel (58.5%), followed by the cost of drugs, which represented 25% of all costs. The median WTP for PrEP was $2 per month (mean was $4.07 per month). This covers only one-third of the monthly cost of the medication (approximately $6 per month) and less than 10% of the full cost of delivering PrEP ($21 per month). A sizable proportion of current clients (27%) were unwilling to pay anything for PrEP. Certain populations put a higher value on PrEP services, including: FSW and MSM, Muslims, individuals with higher education, persons between the ages of 20 and 35, and households with a higher income and expenditures.

**Discussion:**

This is the most recent and comprehensive study on the cost of PrEP delivery in Kenya. These results will be used in determining resource requirements and for resource mobilization to facilitate sustainable PrEP scale-up in Kenya and beyond. This contingent valuation study does have important implications for Kenya's PrEP program. First, it indicates that some populations are more motivated to adopt oral PrEP, as indicated by their higher WTP for the service. MSM and FSW, for example, placed a higher value on PrEP than AGYW. Higher educated individuals, in turn, put a much higher value on PrEP than those with less education (which may also reflect the higher “ability to pay” among those with more education). This suggests that any attempt to increase demand or improve PrEP continuation should consider these differences in client populations. Cost recovery from existing PrEP clients would have potentially negative consequences for uptake and continuation.

## Introduction

Recent evidence suggests that the use of oral pre-exposure prophylaxis (PrEP) is highly effective at lowering the risk of HIV infection (1–4). Several clinical trials reported high efficacy of oral PrEP among individuals at high risk of HIV acquisition, ranging from 99% among men who have sex with men (MSM) to 94% among female sex workers (FSW) (1, 2, 5, 6). Earlier randomized clinical trials reported efficacy ranging from 44%–75% among high-risk individuals in heterosexual relationships, while demonstration projects reported effectiveness greater than 80% (4, 7–9).

In 2015, the World Health Organization recommended that PrEP be offered “as an additional prevention choice for people at substantial risk of HIV infection as part of combination HIV prevention approaches (10).” However, WHO also noted that “PrEP costs are substantial, and include costs for clinic staff, medications, laboratory testing, pharmacy services, community education, provider education and monitoring and evaluation.” They noted that PrEP can be cost saving when the incidence of HIV is greater than 3 per 100 person-years and may still be cost-effective at lower levels of incidence. Kenya included PrEP as part of its combination HIV prevention interventions and subsequently developed guidelines on the use of PrEP (11) in 2017. However, there has been limited information on the cost of scaling up the use of oral PrEP across various populations in the country.

A key component of PrEP adherence relates to how individuals value their medication. Is the medication perceived as being effective, for example? Do individuals have preference for using other prevention methods rather than PrEP (e.g., condoms)? Do certain populations view PrEP as being more valuable than other populations? Understanding how individuals value a good or service, and which service they prefer, can be determined based on their “stated preference.” Stated preferences are frequently utilized when a competitive marketplace does not exist, but there is still a need to understand the magnitude of the benefits that are accrued, as well as the preferences of the consumers. The stated preference approach is most widely used where there is no clear competitive market. In the WTP approach, consumers are asked to state their maximum WTP for a good or service.

For this study, WTP was utilized to understand the perceived benefits of PrEP among members of key populations and adolescent girls and young women (AGYW) in Kenya, as well as to assess the factors that influence a client's strength of preference for PrEP. One objective of this study was to estimate the costs of delivering oral PrEP to various populations through integrated platforms and in different geographic regions of Kenya. A second objective was to determine the preference and strength of preferences for oral PrEP by clients and potential clients in Kenya.

## Methodology

### Source data

A total of 4 data collectors were assigned responsibility for collecting data at each of 44 facilities where PrEP services were available. Each 2-person team then split responsibility between conducting a costing of individual facilities and interviewing individual clients to assess their WTP. Data collection was completed between November 2017 and September 2018.

### Costing

The *Jilinde* (translated “protect yourself”) project (12) was implemented in ten out of the forty-seven counties in Kenya. These counties are classified into three clusters based on geographic proximity: Coast (Mombasa, Kilifi, Kwale and Taita Taveta counties), Nairobi (Nairobi, Kiambu and Machakos counties) and Lake cluster (Kisumu, Kisii and Migori counties). The costed facilities included all facilities where PrEP was being offered by Jilinde, including 23 public health facilities, 16 drop-in-centers and 5 private facilities.

A semi-structured, interviewer-administered questionnaire was used to obtain retrospective cost information from 44 facility managers on the costs of resources used in delivering PrEP services. The data collected included personnel, equipment, medications, consumables, lab tests and reagents, test kits, utility, maintenance, and utilities for each visit (initial visit or first contact, refill visits and quarterly visits) and for each stage of client flow in a facility: (i) Reception, (ii) Triage, (iii) Health education, (iv) HTS and STI testing, (v) Prescription of drugs and (vi) Dispensing drugs client).

### Contingent valuation

The contingent valuation survey was administered to 1,940 PrEP clients and 318 non-PrEP clients. Respondents were interviewed about their personal and household characteristics (age, religion, marital status, individual income, household income, level of education, employment status, etc.). Respondents were asked to self-identify as to whether they were MSM, FSW or AGYW. All participants in the WTP study were asked to sign a consent form noting that they had been explained the purpose of the study, understood that they would not be compensated for participating, and were voluntarily choosing to participate.

Participants were enrolled during their routine visits to these facilities for PrEP and other services. PrEP clients were selected based on their availability during the data collection process and their willingness to be interviewed. Non-PrEP clients were selected based on their utilization of health services at the identified facilities. In some cases, non-PrEP clients were individuals who had been offered PrEP services but had not yet adopted the intervention.

Data from PrEP clients were collected during initial visits (screening visits), refill visits and quarterly visits (13). The initial visits describe the first screening visit, the refill visits describe the Month 2, 4, 5, 7, 8, 10 and 11 where PrEP clients visited the facilities to only refill their PrEP. Quarterly visits were conducted at 3, 6, 9 and 12 months and included an HIV test, clinical assessment, and PrEP refills.

As for the WTP surveys, respondents were reminded that PrEP was being offered for free but asked, “if it was necessary to pay a small amount to participate in this program, would you be willing to do so?” Understanding the factors that influence a person's decision not to pay for a service is critical in assessing if the decision is based on the value of the service (“true zero”), or if it is based on a protest against the idea of paying for any service (“protest zero”) (14). The two most common reasons given for not being willing to pay for PrEP were: (1) since other HIV services are free, PrEP should be free or (2) I have insufficient funds to pay for PrEP. Respondents who indicated that “PrEP services should be free because other prevention methods are free” were categorized as “protest zeros” because the response did not necessarily indicate how they valued PrEP. On the other hand, if a respondent indicated that they did not have enough money to pay for PrEP, it was assumed to be a true reflection of value given their lack of resources and they were thus categorized as a “true zero.” If a respondent did not provide a reason they would be unwilling to pay for PrEP, they were assumed to be a “true zero.” The analysis then focused only on those who indicated a willingness to pay and those who indicated that their unwillingness to pay represented a “true zero.”

If a respondent did indicate a WTP, they were then shown 14 payment cards (including an “other” card), randomly distributed in front of the respondent, with various amounts in Kenyan Shillings. This “payment card approach” has been widely used as it tends to produce high response rates that are closely aligned with an individual's ability to pay, while at the same time avoiding anchoring bias (15). The amounts varied from Kshs 0–Kshs 10,000 (US$100). Respondents were then asked to indicate which card represented the highest amount they would be willing to pay monthly for PrEP services. Once an amount was selected, they were then shown the card with the next highest amount and asked if they would pay that amount. If the respondent indicated “no,” then the original amount selected would be identified as the maximum WTP. If the respondent indicated “yes,” then the next highest card was selected, and they were again asked if they would be willing to pay this amount. In this way, the respondent is “bid up” until they confirm that the amount indicated is truly the maximum WTP. After two attempts to “bid up” the respondent, the bidding process ended, and the highest amount was confirmed.

### Ethical considerations

A protocol was submitted to Kenya Medical Research Institute (KEMRI) in Kenya and the Johns Hopkins School of Public Health (JHSPH) in the US. Approval for the study was received from KEMRI (No. 0583) on October 9, 2017. Institutional review board (IRB) approval was also received from JHSPH (IRB No. 00007657) on January 17th, 2018.

### Statistical analyses

Independence between the WTP and categorical and continuous variables were analyzed, in bivariate analysis, using Fisher exact test, since WTP is discrete by design.

A multivariate polytomous logistic regression model was used to determine the characteristics that are associated with the willingness to pay variable for which we considered the categories 0-to-0.5, 1, 2, and 4-to-100. All variables that were believed to be relevant for the analysis were included in the regression. Stepwise procedure was then used for model selection. Data were analyzed with R version 3.5.1 (16).

## Results

### Cost analysis

The total number of PrEP clients (initial, refill and quarterly visits) for a six-month period preceding data collection (April–September 2017 (phase 1), and from October 2017–March 2018 (phase 2) was 8,256. The Nairobi cluster accounted for 47% of all clients, while the Lake and Coast clusters accounted for 31% and 22% of clients, respectively. The largest number of PrEP clients were FSW (66%), followed by MSM (15%) and then serodiscordant couples (SDC) (13%). General population (GP) and AGYW accounted for 5% and 1%, respectively.

**Figure 1:**
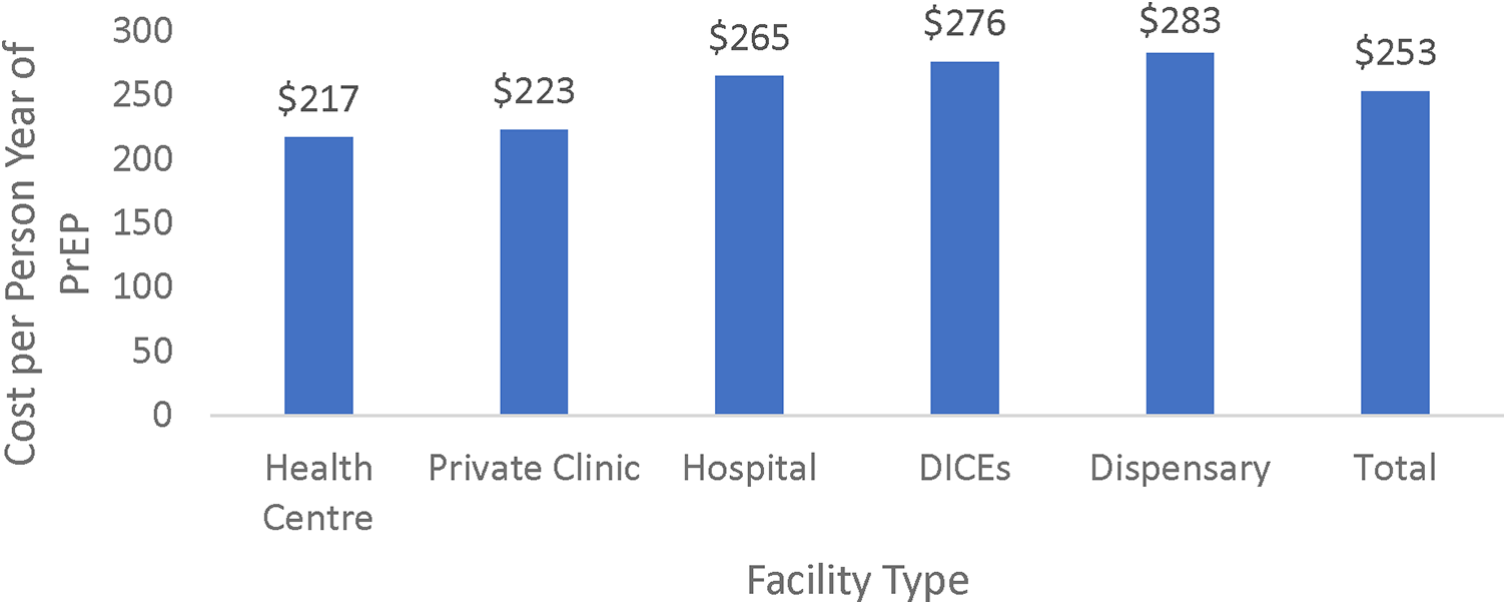
Weighted unit cost—total and by type of facility.

The cost per person year (CPPY) is the cost of providing one client with PrEP services including (generic TDF/FTC) for 12 months. The overall weighted unit CPPY across all the 44 facilities was $253 ranging from $217 at health centres to $283 at dispensaries. The unit cost for DICEs, which served about two-thirds of the client volume at surveyed facilities, was $276. The weighted unit cost of PrEP at the dispensaries was the highest, with a unit cost of $283. These are depicted in Figure 1.

**Figure 2:**
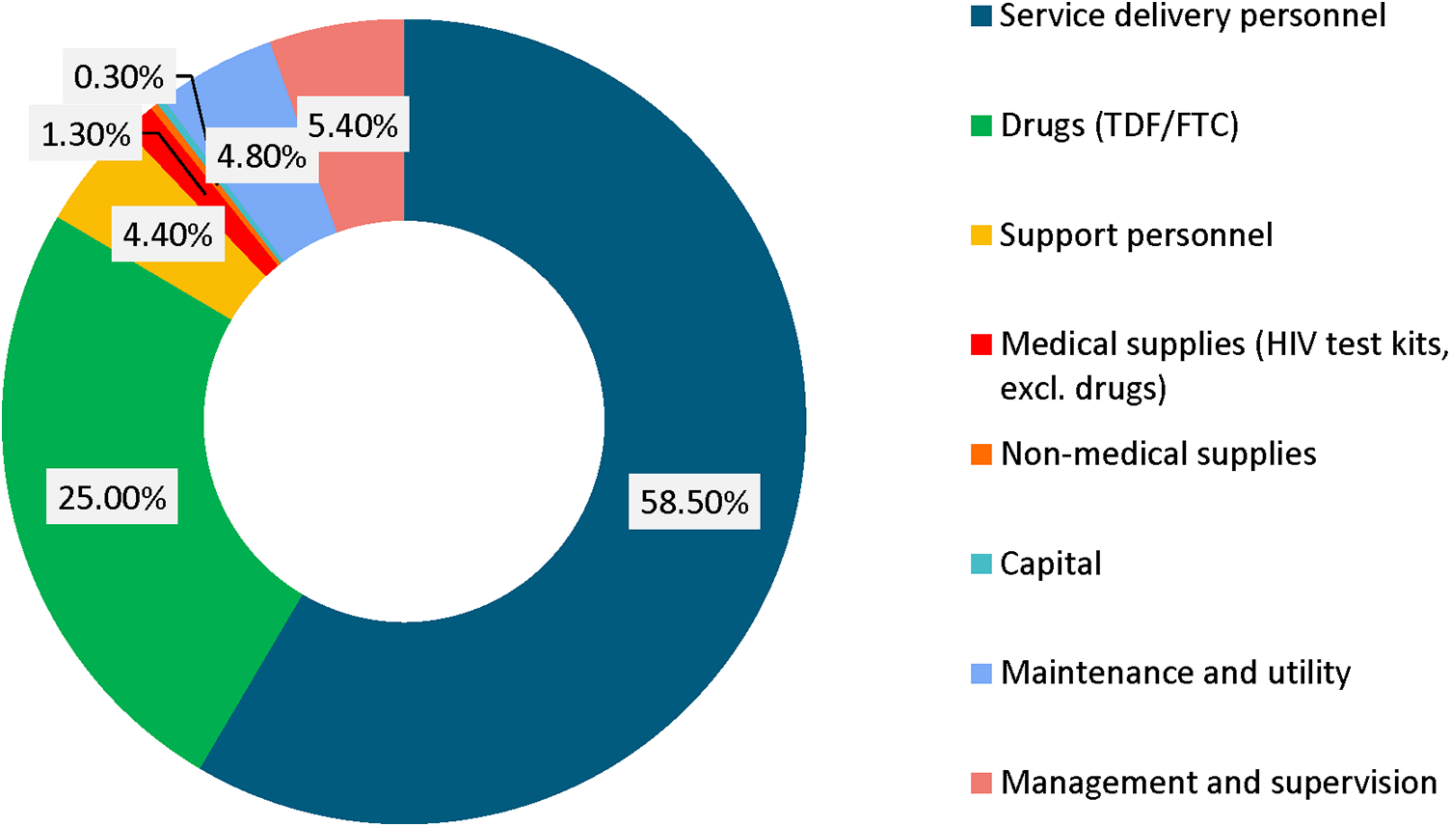
Unit cost by major cost categories.

Examining the unit cost components, the largest was personnel which represented 58.5% of all costs, followed by drugs (generic TDF/FTC), which represented 25% of all costs. The other cost components are illustrated in Figure 2.

**Figure 3:**
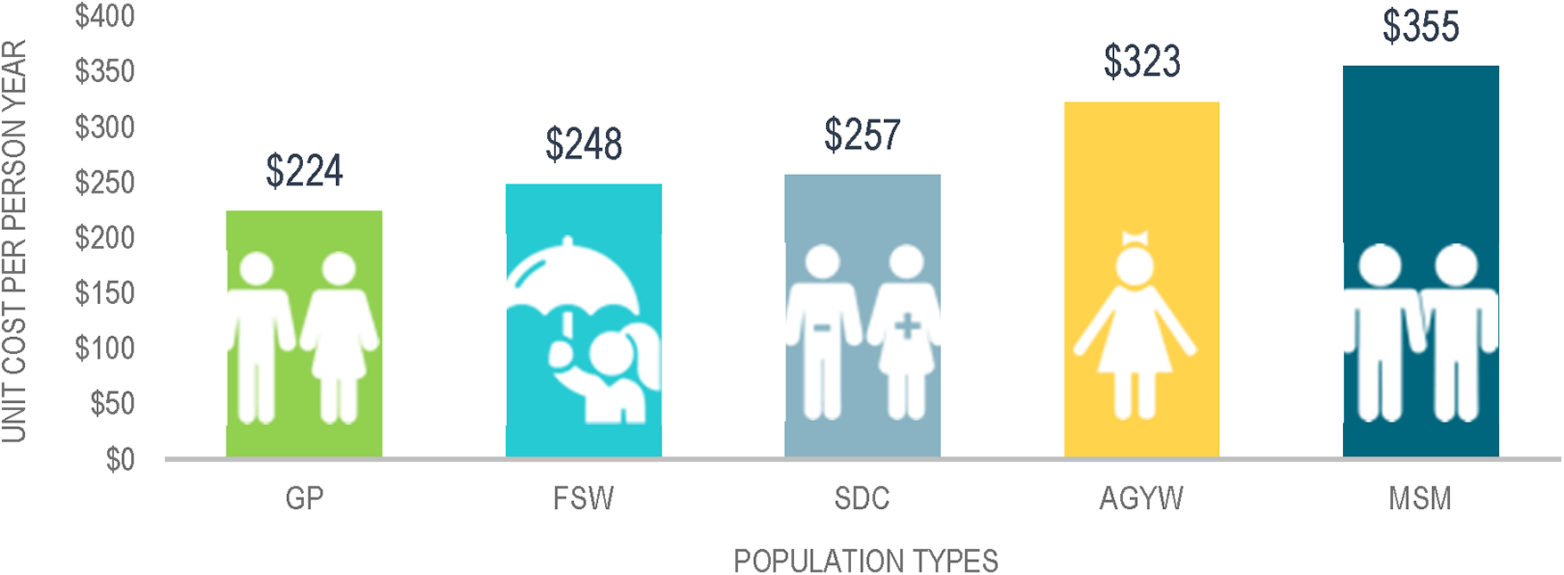
Weighted unit cost per person year by target population.

Regarding the target populations for PrEP, the weighted unit cost per person year on PrEP varied widely from a low of $224 for GP to a high of $355 for MSM. The unit costs per person year for each of the target populations is shown in Figure 3.

Across the three cluster regions, the average weighted cost of PrEP varied marginally. The unit costs of PrEP were higher in the Coast cluster than in the Lake cluster: $269 vs. $267, respectively. Nairobi cluster had the lowest unit cost, estimated at $263. Differences in the unit cost of PrEP by cluster were not statistically significant (Nairobi cluster vs. Lake cluster, *p* = 0.57; Nairobi cluster vs. Coast cluster, *p* = 0.65 and Coast cluster vs. Lake cluster, *p* = 0.59).

### Cost of PrEP per visit

The estimated unit cost for the initial visit varied across service delivery models and by population type. For FSW, the unit cost for the initial visit ranged from $44 in the DICEs to $62 in hospitals. The estimated unit cost for refills was highest at the DICEs ($21 per visit) and lowest at health centers ($12 per visit). The average costs during quarterly visits varied between $34 for the private facilities to $20 for the hospitals. MSM were only served in DICEs and the unit cost was $62 for the initial visit, $22 for the refills and $40 for quarterly visits. For AGYW, the unit cost for the initial visit was higher in private facilities, at $100 compared to $52 in hospitals. The lowest unit cost for refills was in the hospitals ($17) and highest in private facilities ($26). For quarterly visits, the cost per visit was estimated at $32 and $76 in the hospitals and private facilities, respectively. The costs for the various populations for each type of visit are summarized in Table 1.

**Table 1:** Unit costs per visit and per person-year for PrEP (2018 US dollars).

Population	Service delivery	Initial visit	Refills	Quarterly visits	CPPY
FSW	DICEs	$44	$21	$32	$305
	Health center	$48	$12	$28	$229
	Hospital	$62	$20	$20	$324
	Private	$39	$16	$34	$272
	Dispensary				
MSM	DICEs	$62	$22	$40	$349
	Health center				
	Hospital				
	Private				
	Dispensary				
AGYW	DICEs				
	Health center				
	Hospital	$52	$17	$32	$287
	Private	$100	$26	$76	$538
	Dispensary				
GP	DICEs				
	Health center	$51	$17	$37	$271
	Hospital				
	Private				
	Dispensary				
SDC	DICEs				
	Health center	$43	$17	$28	$265
	Hospital	$45	$16	$32	$260
	Private	$34	$15	$26	$233
	Dispensary	$52	$18	$29	$283

By altering the frequency of HIV testing to every six months instead of every three months, the CPPY estimates dropped by between 0%–19%. This varied by population type and service delivery model as depicted in Table 2.

**Table 2:** Variation of CPPY by reducing frequency of HIV testing by population type and service delivery model.

Population	Service delivery model	CPPY (HIV testing every 3 months)	CPPY (HIV testing every 6 months)	% Decrease with 6 m vs. 3 m testing
FSW	DICEs	$308	$286	7.1%
	Health center	$228	$196	14.0%
	Hospital	$282	$282	0.0%
	Private	$269	$233	13.4%
	Dispensary			
MSM	DICEs	$358	$322	10.1%
	Health center			
	Hospital			
	Private			
	Dispensary			
AGYW	DICEs			
	Health center			
	Hospital	$284	$254	10.6%
	Private	$536	$436	18.7%
	Dispensary			
GP	DICEs			
	Health center	$274	$228	16.8%
	Hospital			
	Private			
	Dispensary			
SDC	DICEs			
	Health center	$263	$241	8.4%
	Hospital	$269	$237	11.9%
	Private	$232	$210	9.5%
	Dispensary	$283	$261	7.8%

To better understand the costs associated with integrating PrEP services into public health facilities, we conducted an incremental unit cost analysis. Whereas full economic costing analyses all resources used in the delivery of PrEP, the incremental costing analysis considered the cost of drugs (generic TDF/FTC), medical supplies (HIV test kit, gloves, dry and wet swabs), non-medical supplies (client files) and the cost of provider training in the analysis. Given that PrEP is provided as an additional service for clients in public health facilities, a facility may not require additional staff but rather may undertake training for clinical staff on PrEP delivery.

The average annual incremental cost of delivering PrEP across the different public health facilities was $84 per person per year. The variation of the incremental cost of PrEP delivery between public health facilities was small: $84.80 vs. $83.56 for health centers and hospitals, respectively. The incremental cost in the dispensaries was $83.64 per person per year.

**Figure 4:**
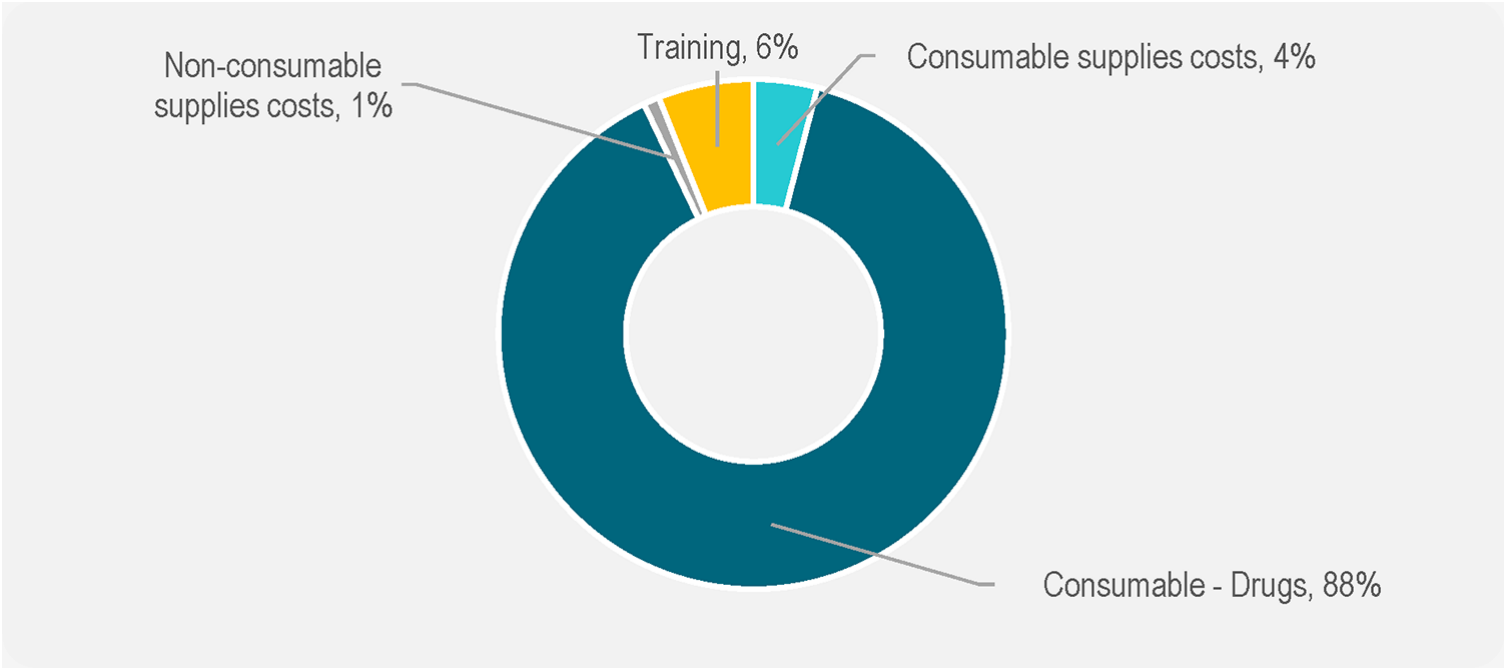
Components of incremental cost of PrEP (%).

The largest component of the incremental costs was drugs (generic TDF/FTC), at $ 75.24 (88% of all the costs), followed by training, at $5.55 (6%) and medical supplies, $3.81 (4%). These are depicted in Figure 4.

### Contingent valuation analysis

A total of 2,258 individuals participated in the survey and reported their WTP for PrEP in Kenya. The characteristics of these respondents are included in Table 3. This included 1,683 female sex workers (74.5%), 308 men who have sex with men (13.6%), 264 adolescent girls and young women (11.7%) and 3 who were not classified (0.1%). Of the respondents, 989 were in the Lake cluster (43.8%), 764 were in the Nairobi cluster (33.8%) and 505 were in the Coast cluster (22.4%). Respondents tended to be young, with the majority (51.8%) being under the age of 25. Most individuals were single and had never been married (61.3%). Most of the respondents have had at least some high school education (64%), with 13% having at least some tertiary education. The most common religion reported by respondents was Protestant Christians, who represented 60% of the sample size. Of the total population of respondents, 86% were current PrEP clients, while the remaining 14% were members of a key population or AGYW but were not currently PrEP clients.

**Table 3:** Descriptive characteristics of the full sample.

Variable		% (*N* = 2,258)
Population		
	AGYW	11.69%
	FSW	74.53%
	MSM	13.64%
	Missing	0.13%
Cluster		
	Central Region	28.57%
	Coastal Region	22.36%
	Lake Region	43.80%
	Nairobi	5.27%
Age		
	15–19	11.65%
	20–24	33.75%
	25–34	41.14%
	35+	13.42%
	Missing	0.04%
Marital status		
	Living together	1.77%
	Married/in union	7.71%
	Never married/Single	61.20%
	Widowed/separated/divorced	29.23%
	Missing	0.09%
Level of education		
	College/Higher/Tertiary(completed)	8.59%
	College/Higher/Tertiary(not completed)	4.47%
	No Grade completed(none)	1.51%
	Primary complete	20.24%
	Primary incomplete	13.86%
	Secondary complete	24.93%
	Secondary incomplete	26.35%
	Missing	0.04%
Religion		
	Muslim	7.79%
	No Religion	3.32%
	Protestant/Other Christian	60.14%
	Roman Catholic	28.48%
	Missing	0.27%
Agreed to join PrEP		
	No	13.99%
	Yes	85.92%
	Missing	0.09%
Health insurance		
	No	78.65%
	Yes	17.23%
	Missing	4.12%

Of the total population of respondents, 43% indicated that they would not be willing to pay for PrEP. This differed between current PrEP clients (41%) and non-PrEP clients (50%) (*p* < 0.01). This higher willingness to pay for PrEP by current clients is not surprising, since PrEP clients are already motivated to take PrEP and therefore would be expected to be more incentivized to pay something for PrEP than those who are not already a part of the intervention.

Among existing PrEP clients, respondents were asked if they would pay anything for PrEP. A total of 1,563 respondents indicated either that they had some willingness to pay for PrEP or, if they were unwilling to pay anything, this represented a true valuation of the service.

The median WTP (including those categorized as “true zeros” but excluding those who were “protest zeros”) was US$2 per month. In other words, about half (49%) of all current PrEP clients were willing to pay $2 per month or more for PrEP, while the other half were not willing to pay this much. The mean WTP was $4.07.

The cost to the government of Kenya for a 30-day supply of generic Truvada was approximately $6 per month. As the willingness to pay for PrEP drops off rapidly as the proposed price increases, only about 17% of all clients indicated they would be willing and able to pay this price for PrEP monthly. Only about 5% of all clients were willing to pay $20 per month, which would represent full cost recovery (including the medication, labor, lab tests, etc.).

Table 4 indicates that several categorical variables were associated with willingness to pay. By client population, MSM had the highest mean WTP, at $8.26 per month. This was followed by FSW, who indicated a mean WTP of $3.79 per month. The lowest mean WTP was among AGYW, who indicated a WTP of only $1.36 per month. This is also consistent with data on monthly household income, which indicated that MSM had the highest income ($300 per month), followed by FSW ($255 per month), and AGYW ($90 per month).

**Table 4:** Bivariate analysis of willingness to pay.

Variable		Mean amount WTP (Sd)	*P* value of Fisher's exact test^a^
Population			*P* < 0.01
	AGYW	1.36 (1.12)	
	FSW	3.79 (5.7)	
	MSM	8.26 (16.69)	
	No response	20.5 (27.58)	
Cluster			*P* < 0.01
	Central Region	5.9 (11.8)	
	Coastal Region	6.55 (8.22)	
	Lake Region	2.02 (2.1)	
	Nairobi	2.52 (1.8)	
Age			*P* < 0.01
	15–19	2.51 (3.48)	
	20–24	4.27 (8.42)	
	25–34	4.5 (8.44)	
	35+	3.26 (5.31)	
Marital status			*P* = 0.01
	Living together	9.58 (23.01)	
	Married/in union	3.28 (7.7)	
	Never married/Single	4.18 (7.86)	
	Widowed/separated/divorced	3.66 (5.12)	
	Others	6. (.)	
Level of education			*P* < 0.01
	None/Some Primary/Primary Complete	3.65 (7.64)	
	Some Secondary/Secondary Complete	4.11 (7.99)	
	Some Tertiary/Tertiary Complete	4.97 (7.08)	
	No response	6. (.)	
Religion			*P* < 0.01
	Muslim	7.96 (13.86)	
	No Religion	3.67 (3.09)	
	Protestant/Other Christian	3.55 (6.42)	
	Roman Catholic	4.14 (8.09)	
	No response	4. (.)	
Health insurance			*P* < 0.01
	No	3.7 (6.22)	
	Yes	5.67 (10.65)	
	No response	3.99 (16.72)	
Facility category			*P* < 0.01
	Clinic	1.48 (1.36)	
	DICES	4.41 (8.36)	
	Dispensary	5.78 (9.39)	
	Health center	2.99 (4.43)	
	Hospital	2.96 (4.62)	
Size of the HH			*P* < 0.01
	Q1: 1.0–2.0	4.91 (9.09)	
	Q2: 2.0–3.0	4.2 (8.14)	
	Q3: 3.0–4.0	3.19 (4.87)	
	Q4: 4.0–12.0	2.59 (3.24)	
	No response	11.75 (8.8)	
#Working days/week			*P* < 0.01
	Q1: 0.5–4.0	5.57 (8.51)	
	Q2: 4.0–6.0	4.17 (8.02)	
	Q3: 6.0–7.0	2.68 (3.45)	
	No response	4.5 (13.31)	
Total income			*P* < 0.01
	Q1: 8.0–137.0	3.23 (7.49)	
	Q2: 137.0–200.0	2.86 (4.46)	
	Q3: 200.0–300.0	4.66 (6.79)	
	Q4: 300.0–1,950.0	6.31 (9.93)	
	No Response	3.07 (10.38)	
Total expenditure			*P* < 0.01
	Q1: 2.0–104.8	3.43 (7.72)	
	Q2: 104.8–165.0	3.22 (4.73)	
	Q3: 165.0–252.1	3.81 (6.13)	
	Q4: 252.1–1,244.5	6.32 (9.71)	
	No Response	3.07 (10.27)	

Statistics of the test are not available for Fisher test; simulations with 10,000 samples were used to estimate its *p*-values.

^a^ Continuous variables were discretized following their quartiles, and WTP is discrete by design.

Breaking down the mean monthly WTP by cluster, respondents in the Lake cluster were willing to pay much less ($2.02) than in either Coast ($6.55) or Nairobi ($2.52) clusters. This is consistent with data regarding household income, which indicates that monthly household income in the Lake Cluster ($176 per month) is much lower than in either the Coast ($305 per month) or Nairobi ($307 per month) clusters.

Based on the location where PrEP clients receive services, clients at dispensaries had the highest WTP ($5.78 per month). This was then followed by DICES ($4.41 per month), Health Centers ($2.99 per month), Hospitals ($2.96 per month) and clinics ($1.48 per month). This result might either be an indication that those who attend DICEs or Dispensaries (mostly FSW and MSM) are the most motivated to obtain PrEP, or alternatively it may mean that those who attend DICEs or dispensaries are the most satisfied with the services they are receiving and therefore are willing to pay more for the services they receive.

WTP also varied by the relationship status of the individuals. The highest mean WTP was for those who were living together but not married ($9.58 per month). This was followed by those who were never married/single ($4.18 per month) and those who were widowed/separated/divorced ($3.66 per month). Those with the lowest willingness to pay were those who were married/in-union ($3.28 per month).

In terms of age, willingness to pay was highest among those 25–34 years old ($4.50 per month). Those who were younger or older than this age range were willing to pay less, with mean WTP varying between $2.51 and $4.27 per month.

In addition, several other variables were also found to be correlated to WTP. These include total monthly income, size of household and total expenditures. As expected, there was a positive relationship between WTP and household monthly income. Individuals who had a higher household income also had a higher willingness to pay. On average, respondents indicated that they would be willing to pay 1.5% of their household income for PrEP. MSM were willing to pay 2.0%, AGYW were willing to pay 2.1% and FSW were willing to pay 1.2%. Thus, relative to their income, AGYW were willing to pay the most while FSW were willing to pay the least.

Several other variables were not found to be statistically significant in terms of WTP. This includes the number of days that the respondent worked in a typical week.

Next, a multivariate regression analysis was performed to determine the variables that are most likely to be associated to an individual's WTP for PrEP. The key variables in the multivariate analysis included the 12 variables that were statistically significant in the univariate analysis:

• The population type of the respondent (MSM, FSW, AGYW)
• The location of the respondent (Lake, Nairobi and Central, Coast)
• The age of the respondent
• The marital status of the respondent
• The education level of the respondent
• The religion of the respondent
• The insurance status of the respondent
• The type of facility where respondent received PrEP
• The size of the household
• The number of days worked in a week
• The income of the respondent
• The total expenditure of the respondent

In assessing the association between variables and then performing a stepwise multinomial regression, only the location, the population type, age, level of education, and total income of the respondent were included. The highest WTP was among MSM and FSWs, those in the Coastal cluster, between the ages of 25 and 34, and those with a higher income and education.

## Discussion

The analysis showed that overall, it costs $253 per person year on PrEP in Kenya, with variability between the different service delivery models and the populations served. The variability in the cost estimates could be explained by the delivery approach (whether it was through outreach or through static facilities), the volume of PrEP clients, and the number of personnel involved in the delivery pathway. As the scale-up continues, the unit costs are likely to be reduced due to economies of scale and increased efficiencies in delivery.

This contingent valuation study does have important implications for Kenya's PrEP program. First, it indicates that some populations are more motivated to adopt oral PrEP, as indicated by their higher WTP for the service. MSM and FSW, for example, placed a higher value on PrEP than AGYW. Higher educated individuals, in turn, put a much higher value on PrEP than those with less education (which may also reflect the higher “ability to pay” among those with more education). This suggests that any attempt to increase demand or improve PrEP continuation should consider these differences in client populations. Cost recovery from existing PrEP clients would have potentially negative consequences for uptake and continuation.

### Cost

The key cost drivers across all service delivery models were personnel and drug costs (generic TDF/FTC) which accounted for over 80% of the weighted unit costs. This is consistent with earlier studies, which found that the two primary cost drivers associated with providing PrEP are personnel and drugs (17–19). Other noticeable cost drivers were management and supervision, and support personnel. These findings suggest that any effort to reduce PrEP costs should focus on leveraging the existing personnel by integrating PrEP into routine services. This is supported by results from this study which show that the incremental cost of layering PrEP into the existing Ministry of Health facilities would be $84 per person per year, assuming the facility staff absorbs Prep responsibilities. These results show that the additional cost required to offer PrEP in public health facilities is almost a third the full economic costs. These findings are comparable with a study on integrating PrEP into routine maternal and child health and family planning in Western Kenya which found that drugs accounted for 25% of the total programme costs (19). Based on these results, sustainability could be guaranteed by ensuring consistency in supply of PrEP commodities, complemented with minimal resources for provider training, medical supplies, and management and supervision.

The study found differences in the unit cost depending on the type of visit, with the highest costs estimated for the initial visit, followed by the quarterly visit and then the refill visit. There was variability in the cost for the three visits across service delivery models and population type. These variations could be accounted for by the explanations on the overall unit cost, and our findings are consistent with other studies conducted in Africa (20–22). The unit cost for the initial visit is much higher compared to the unit costs for refills and quarterly visits. This difference is because clients spend more time with providers during initiation and quarterly visits on HIV testing, adherence counselling and eligibility assessment, compared to refills, where most of the time is spent at the dispensing points. This is supported by time and motion studies (18) which found the time taken to conduct activities related to PrEP and ART for discordant couples was 42 min during screening/initial visits and 36 min during follow-up visits.

Our analysis suggests that performing HIV testing once every six months could reduce the cost per person per year by up to 19%. While mathematical modelling suggests reducing the frequency of HIV testing could lead to more HIV drug resistance from PrEP implementation (23), a modelling study in South Africa found that quarterly and biannual HIV testing would have a similar health impact and resistance consequences (24). Other innovative approaches that could reduce costs by reducing frequency of clinic visits and increasing efficiency include differentiated PrEP delivery models. For instance, dispensing intervals can be increased by using multi-month dispensing for clients who have demonstrated good adherence, to reduce the number of refill visits. Additionally, group refills and counselling which have been successfully applied in antiretroviral therapy and antenatal care settings, can also be explored (25). Further research is warranted on the feasibility of using HIV self-testing to empower clients to monitor their HIV status while on PrEP and reduce the intensity of clinic-based monitoring.

### Contingent valuation

The contingent valuation study determined that the average willingness to pay was insufficient to cover the cost of the medications, not to mention the full cost of delivering PrEP in Kenya. Any attempt to achieve cost recovery from the existing populations would likely result in significantly reduced demand and reduced continuation rates among those who have adopted PrEP. On the other hand, the results may suggest an opportunity to use financial incentives to encourage greater use of PrEP. Additional experimental research may establish the impact on uptake and continuation if small incentives were introduced.

While contingent valuation studies are useful for assessing if cost recovery is or is not feasible, they also provide critical information about how different populations value goods and services. In this case, the study found that certain populations place a higher value on PrEP than others, as indicated by their higher willingness to pay. AGYW, for example, placed a lower value on PrEP than MSM or FSW, but had a higher WTP relative to their income. Higher educated individuals and higher income individuals, in turn, put a much higher value on PrEP. Individuals from Nairobi and Coast clusters placed a higher value on PrEP than individuals from the Lake cluster. In terms of age, the highest valuation peaked among those who were 25–34 years old. Those who received PrEP at dispensaries had a higher WTP. This suggests that any attempt to increase demand or improve PrEP continuation should consider these differences in client populations.

There are various potential explanations for why certain populations have a higher WTP than others. The lower WTP in the Lake cluster may reflect existing intensive and free HIV prevention programming in the region, which might cause the respondents to indicate a lower WTP than those in the other two clusters where access to free services might be more limited. The data on WTP among AGYW relative to income may be an indication that adolescents have fewer other financial burdens (e.g., rent and food might already be paid by parents or other relatives), and therefore they are willing to pay a greater percentage of their income on services such as PrEP. The fact that WTP peaks at 25–34 years of age may reflect either higher income at that age, or higher risk.

Contingent valuation is one tool that is available that assists researchers and policymakers to examine the motivations of consumers as they value a health service such as PrEP. Understanding what motivates PrEP clients requires more than an understanding of personal costs, gains, and risk. As indicated in previous research, factors such as social capital are also critical in understanding how individuals can be recruited into PrEP programs and enabled to remain on PrEP (26).

In conclusion, the two components of this study found useful findings. The costing study confirmed the cost of oral PrEP in Kenya, while also noting how costs may vary from site to site. The contingent valuation study found that there are a range of factors that influence the value placed on PrEP by clients.

### Study limitations

There are various limitations, which should be considered as part of this study. First, the cost analysis was based on sites located in certain counties in Kenya. These sites may not necessarily be representative of other counties throughout the country.

In addition, the costing exercise was based on interviews with providers and did not entail following clients. Therefore, the time spent with clients was based on the responses detailed by the providers and not based on actual observed provision of services. This may entail some bias, as providers may either underestimate over overestimate the actual time spent with clients.

Next, the costing categorized sites based on the primary target population of each site and not based on individual clients. This may also introduce bias, as the total cost at facilities may not be driven by the targeted clients, but rather may be influenced by other populations that are reached.

In terms of the contingent valuation study, this requires an accurate assessment of valuation by PrEP clients. However, there are several reasons why individuals may not reveal their true maximum WTP. On one end, respondents may understate their WTP, to avoid providing any information that might lead to a higher price being charged for the service being discussed. On the other hand, respondents may overstate their WTP. Respondents, for example, might want to indicate that they are enthusiastic about continuing with the intervention, even if they are not truly capable of paying the indicated amount monthly.

Another limitation to this study concerns the “protest zero” responses. The authors attempted, to the best of their ability, to distinguish between: (1) those who indicated truly that they were unwilling or unable to pay for PrEP, and (2) those who responded that they were unwilling to pay for PrEP based on a protest towards the idea of having to pay for PrEP. Distinguishing between these two types of responses was problematic. If some of the responses categorized as “protest zeros” (and therefore excluded from the analysis) were “true zeros,” then the average WTP estimates may be overestimated. Conversely, if some of the “true zeros” really represented protests to the idea of having to pay (and therefore should have been excluded), then the average WTP may have been underestimated.

## Data Availability

The raw data supporting the conclusions of this article will be made available by the authors, without undue reservation.

## References

[1] Fonner VA Dalglish SL Kennedy CE Baggaley R O’Reilly KR Koechlin FM Effectiveness and safety of oral HIV preexposure prophylaxis for all populations. AIDS. (2016) 30(12):1973–83. 10.1097/QAD.0000000000001145 27149090 PMC4949005

[2] McCormack S Dunn DT Desai M Dolling DI Gafos M Gilson R Pre-exposure prophylaxis to prevent the acquisition of HIV-1 infection (PROUD): effectiveness results from the pilot phase of a pragmatic open-label randomised trial. Lancet. (2016) 387(10013):53–60. 10.1016/S0140-6736(15)00056-2 26364263 PMC4700047

[3] Baeten JM Donnell D Ndase P Mugo NR Campbell JD Wangisi J Antiretroviral prophylaxis for HIV prevention in heterosexual men and women. N Engl J Med. (2012) 367(5):399–410. 10.1056/NEJMoa1108524 22784037 PMC3770474

[4] Grant RM Lama JR Anderson PL McMahan V Liu AY Vargas L Preexposure chemoprophylaxis for HIV prevention in men who have sex with men. N Engl J Med. (2010) 363:2587–99. 10.1056/NEJMoa1011205 21091279 PMC3079639

[5] Anderson PL Glidden DV Liu A Buchbinder S Lama JR Guanira JV Emtricitabine-tenofovir concentrations and pre-exposure prophylaxis efficacy in men who have sex with men. Sci Transl Med. (2012) 4(151):1–17. 10.1126/scitranslmed.3004006 PMC3721979 22972843

[6] Landovitz RJ. Preexposure prophylaxis for hiv prevention: what we know and what we still need to know for implementation. Top Antivir Med. (2015) 23(2):85–90. 26200708 PMC6148938

[7] Thigpen MC Kebaabetswe PM Paxton LA Smith DK Rose CE Segolodi TM Antiretroviral preexposure prophylaxis for heterosexual HIV transmission in Botswana. N Engl J Med. (2012) 367(5):423–34. 10.1056/NEJMoa1110711 22784038

[8] Choopanya K Martin M Suntharasamai P Sangkum U Mock PA Leethochawalit M Antiretroviral prophylaxis for HIV infection in injecting drug users in Bangkok, Thailand (the Bangkok Tenofovir study): a randomised, double-blind, placebo-controlled phase 3 trial. Lancet. (2013) 381:2083–90. 10.1016/S0140-6736(13)61127-7 23769234

[9] Antoni G Tremblay C Delaugerre C Charreau I Cua E Rojas Castro D On-demand pre-exposure prophylaxis with tenofovir disoproxil fumarate plus emtricitabine among men who have sex with men with less frequent sexual intercourse: a post-hoc analysis of the ANRS IPERGAY trial. Lancet HIV. (2020) 7(2):e113–20. 10.1016/S2352-3018(19)30341-8 31784343

[10] World Health Organization. Guideline on When To Start Antiretroviral Therapy and on Pre-Exposure Prophylaxis for HIV. Geneva, Switzerland: WHO (2015). 26598776

[11] NASCOP. Framework for the Implementation of Pre-Exposure Prophylaxis of HIV In Kenya. Vol. 96, NASCOP. Nairobi, Kenya (2017). Available online at: https://www.prepwatch.org/wp-content/uploads/2017/05/Kenya_PrEP_Implementation_Framework.pdf (accessed September 9, 2022).

[12] Were D Musau A Mugambi M Plotkin M Kabue M Manguro G An implementation model for scaling up oral pre-exposure prophylaxis in Kenya: Jilinde project. Gates Open Research. (2021) 5:1–9. 10.12688/gatesopenres.13342.1 PMC8669463 34988373

[13] Ministry of Health NA& SCP (NASCOP). Guidelines on Use of Antiretroviral Drugs for Treating and Preventing HIV in Kenya. Nairobi, Kenya (2018). Available online at: https://www.nascop.or.ke/new-guidelines/ (accessed May 19, 2022).

[14] Frey UJ Pirscher F. Distinguishing protest responses in contingent valuation: a conceptualization of motivations and attitudes behind them. PLoS One. (2019) 14(1):1–20. 10.1371/journal.pone.0209872 PMC6324805 30620731

[15] Donaldson C. Willingness to Pay for Publicly-Provided Health Care. Aberdeen: University of Aberdeen (1996).

[16] R Foundation for Statistical Computing. R: a Language and Environment for Statistical Computing (2018). Available online at: http://www.R-project.org/

[17] Suraratdecha C Stuart RM Manopaiboon C Green D Lertpiriyasuwat C Wilson DP Cost and cost-effectiveness analysis of pre-exposure prophylaxis among men who have sex with men in two hospitals in Thailand. J Int AIDS Soc. (2018) 21(S5):39–46. 10.1002/jia2.25129 PMC6055129 30033559

[18] Irungu EM Sharma M Maronga C Mugo N Ngure K Celum C The incremental cost of delivering PrEP as a bridge to ART for HIV serodiscordant couples in public HIV care clinics in Kenya. AIDS Res Treat. (2019) 2019:3. 10.1155/2019/4170615 PMC6521338 31186955

[19] Roberts DA Barnabas R V Abuna F Lagat H Kinuthia J Pintye J The role of costing in the introduction and scale-up of HIV pre-exposure prophylaxis: evidence from integrating PrEP into routine maternal and child health and family planning clinics in western Kenya. J Int AIDS Soc. (2019) 22(S4):71–7. 10.1002/jia2.25296/full PMC6643078 31328443

[20] Eakle R Gomez GB Naicker N Bothma R Mbogua J Cabrera Escobar MA HIV pre-exposure prophylaxis and early antiretroviral treatment among female sex workers in South Africa: results from a prospective observational demonstration project. PLoS Med. (2017) 14(11):1–17. 10.1371/journal.pmed.1002444 PMC5697804 29161256

[21] Chen A Kosimbei G Mwai D. Cost of Providing Oral Pre-Exposure Prophylaxis To Prevent Hiv Infection Among Sex Workers in Kenya (2014) (August):1–20. Available online at: https://www.healthpolicyproject.com/pubs/210_KenyaOralPrEPReportFINAL.pdf (cited May 10, 2023).

[22] Cremin I McKinnon L Kimani J Cherutich P Gakii G Muriuki F PrEP for key populations in combination HIV prevention in Nairobi: a mathematical modelling study. Lancet HIV. (2017) 4(5):e214–22. 10.1016/S2352-3018(17)30021-8 28233660

[23] Abbas UL Glaubius R Mubayi A Hood G Mellors JW. Antiretroviral therapy and pre-exposure prophylaxis: combined impact on HIV transmission and drug resistance in South Africa. J Infect Dis. (2013) 208(2):224–34. 10.1093/infdis/jit150 23570850 PMC3895950

[24] Phillips AN Cambiano V Johnson L Nakagawa F Homan R Meyer-Rath G Potential impact and cost-effectiveness of condomless-sex-concentrated PrEP in KwaZulu-Natal accounting for drug resistance. J Infect Dis. (2021) 223(8):1345–55. 10.1093/infdis/jiz667 31851759 PMC8064039

[25] Prust ML Banda CK Nyirenda R Chimbwandira F Kalua T Jahn A Multi-month prescriptions, fast-track refills, and community ART groups: results from a process evaluation in Malawi on using differentiated models of care to achieve national HIV treatment goals. J Int AIDS Soc. (2017) 20(Suppl 4):41–50. 10.7448/IAS.20.5.21650 PMC5577715 28770594

[26] Salas E Fiore S Warner N. This reading is protected under the copyright law of the United States. The copyright law of the United States (Title 17 U. S. Code) governs the making of photocopies or other reproductions of copyrighted material. Copying, displaying and distribut).

